# Illness Perceptions of COVID-19 in Europe: Predictors, Impacts and Temporal Evolution

**DOI:** 10.3389/fpsyg.2021.640955

**Published:** 2021-04-14

**Authors:** David Dias Neto, Ana Nunes da Silva, Magda Sofia Roberto, Jelena Lubenko, Marios Constantinou, Christiana Nicolaou, Demetris Lamnisos, Savvas Papacostas, Stefan Höfer, Giovambattista Presti, Valeria Squatrito, Vasilis S. Vasiliou, Louise McHugh, Jean-Louis Monestès, Adriana Baban, Javier Alvarez-Galvez, Marisa Paez-Blarrina, Francisco Montesinos, Sonsoles Valdivia-Salas, Dorottya Ori, Raimo Lappalainen, Bartosz Kleszcz, Andrew Gloster, Maria Karekla, Angelos P. Kassianos

**Affiliations:** ^1^ISPA - Instituto Universitário, Lisboa, Portugal; ^2^Applied Psychology Research Center Capabilities & Inclusion, Lisboa, Portugal; ^3^Faculdade de Psicologia, Universidade de Lisboa, Lisboa, Portugal; ^4^Psychological Laboratory, Faculty of Public Health and Social Welfare, Riga Stradiṇš University, Riga, Latvia; ^5^Department of Social Sciences (Cyprus), School of Humanities and Social Sciences, University of Cyprus, Nicosia, Cyprus; ^6^Department of Nursing (Cyprus), Cyprus University of Technology, Limassol, Cyprus; ^7^Department of Health Sciences, European University Cyprus, Nicosia, Cyprus; ^8^The Cyprus Institute of Neurology and Genetics, The University of Nicosia Medical School, Nicosia, Cyprus; ^9^Medical University Innsbruck, Innsbruck, Austria; ^10^Department of Human and Social Sciences, Kore University Behavioral Lab (KUBeLab), Kore University of Enna, Enna, Italy; ^11^Kore University Behavioral Lab (KUBeLab), Faculty of Human and Social Sciences, Kore University of Enna, Enna, Italy; ^12^School of Applied Psychology, University College Cork, Cork, Ireland; ^13^School of Psychology (Ireland), University College Dublin, Dublin, Ireland; ^14^LIP/PC2S, University Grenoble Alpes, Grenoble, France; ^15^Department of Psychology, Babes-Bolyai University, Cluj-Napoca, Romania; ^16^Department of Biomedicine, Biotechnology and Public Health, University of Cadiz, Cádiz, Spain; ^17^Instituto ACT, Madrid, Spain; ^18^Department of Psychology, European University of Madrid, Madrid, Spain; ^19^Department of Psychology and Sociology, University of Zaragoza, Zaragoza, Spain; ^20^Vadaskert Child and Adolescent Psychiatric Hospital, Budapest, Hungary; ^21^Department of Psychology, University of Jyväskylä, Jyväskylä, Finland; ^22^Private Practice, Warsaw, Poland; ^23^Division of Clinical Psychology & Intervention Science, Department of Psychology, University of Basel, Basel, Switzerland; ^24^Department of Psychology, University of Cyprus, Nicosia, Cyprus; ^25^Department of Applied Health Research, University College London (UCL), London, United Kingdom

**Keywords:** illness perceptions, COVID-19, common sense model, illness representations, stress

## Abstract

**Objective:** Illness perceptions (IP) are important predictors of emotional and behavioral responses in many diseases. The current study aims to investigate the COVID-19-related IP throughout Europe. The specific goals are to understand the temporal development, identify predictors (within demographics and contact with COVID-19) and examine the impacts of IP on perceived stress and preventive behaviors.

**Methods:** This was a time-series-cross-section study of 7,032 participants from 16 European countries using multilevel modeling from April to June 2020. IP were measured with the Brief Illness Perception Questionnaire. Temporal patterns were observed considering the date of participation and the date recoded to account the epidemiological evolution of each country. The outcomes considered were perceived stress and COVID-19 preventive behaviors.

**Results:** There were significant trends, over time, for several IP, suggesting a small decrease in negativity in the perception of COVID-19 in the community. Age, gender, and education level related to some, but not all, IP. Considering the self-regulation model, perceptions consistently predicted general stress and were less consistently related to preventive behaviors. Country showed no effect in the predictive model, suggesting that national differences may have little relevance for IP, in this context.

**Conclusion:** The present study provides a comprehensive picture of COVID-19 IP in Europe in an early stage of the pandemic. The results shed light on the process of IP formation with implications for health-related outcomes and their evolution.

## Introduction

The new SARS-Cov-2 coronavirus disease (COVID-19) has become the most serious global pandemic in modern times. It has called the attention of our communities to infectious diseases that had seemed controlled in the eyes of the public and led governments to take drastic measures. Among these were the promotion of preventive measures (e.g., hand-washing, social distancing) that require behavior change in daily habits. The need for such widespread behavior changes calls for the understanding of its determinants. This is important, as the level of adherence of the community to these measures should impact the course (e.g., new waves of cases) and severity of the pandemic.

**Figure 1:**
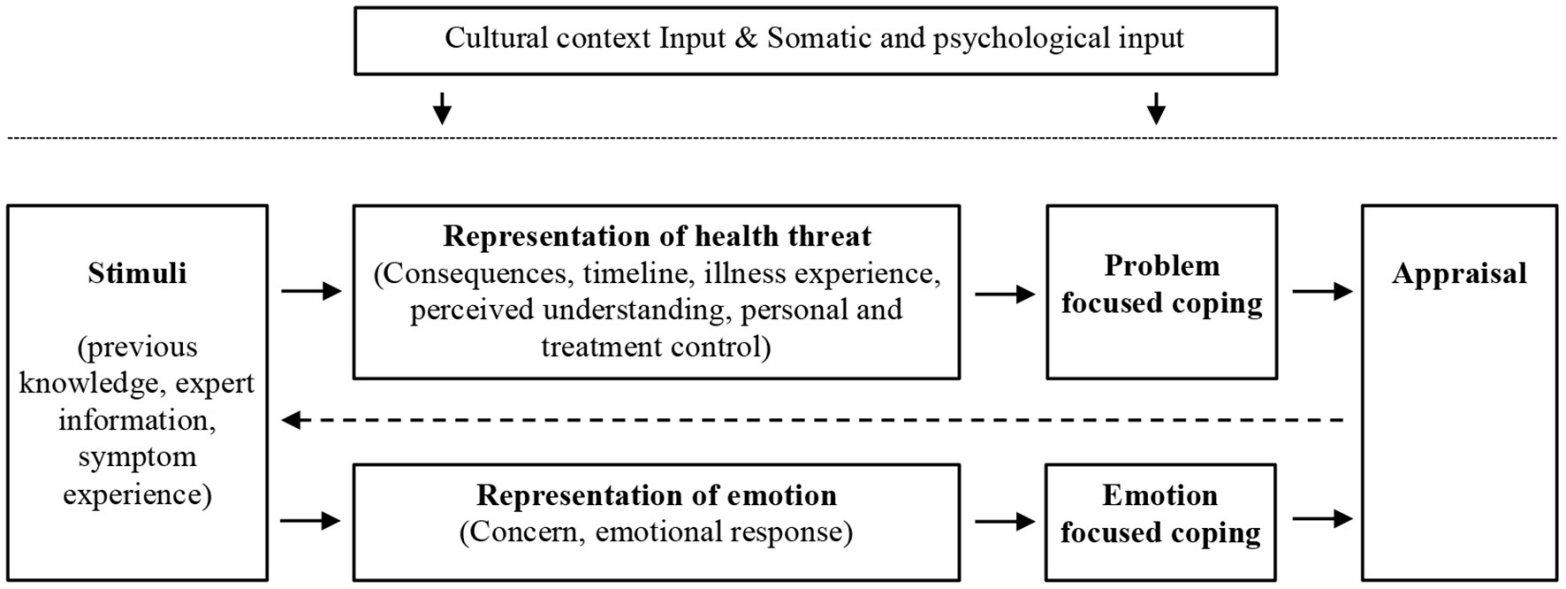
Common-sense model of self-regulation. Adapted from Diefenbach and Leventhal (1996).

The way people perceive illness is one of the relevant factors to understand the adoption of preventive and health management behaviors. Illness perceptions are cognitive representations of disease present in both patients and healthy individuals. The most widely researched theoretical formulation of these representations is based on Leventhal and colleagues' model of self-regulation (Diefenbach and Leventhal, 1996; Leventhal et al., 2003, 2016) - see Figure 1. They proposed that illness perceptions are grouped in different but interrelated components. These components have been classified as cognitive or emotional illness representations (Broadbent et al., 2006). The cognitive representations include perceptions about (a) the consequences of a particular illness, (b) the expected timeline or duration of the illness, (c) personal control of aspects of the disease, (d) the extent of usefulness of treatment in controlling or managing the illness, (e) the perception of the experience of an illness, and (f) its symptoms and understanding or being knowledgeable of the disease. The emotional representations focus on the following: (g) concern or worry about the disease or its consequences, and (h) the emotional response (e.g., fear, anger, and distress) associated with the illness. Illness perceptions are generated by situational stimuli such as symptoms or health information and are assumed to influence coping (Weinman et al., 1996; Broadbent et al., 2006).

With research spanning over 40 years, hundreds of studies and dozens of meta-analyses have been conducted on illness perceptions. Most studies on illness perceptions have been conducted with clinical samples, and consistent associations have been found with help-seeking behaviors and service usage (Baines and Wittkowski, 2013), fear of recurrence of the disease (e.g., breast cancer), quality of life, mood (Foxwell et al., 2013; Kaptein et al., 2015; Rijken et al., 2020), and stress (Karademas et al., 2009; Westbrook et al., 2016; Zhang et al., 2016).

Some conflicting results have emerged concerning the predictive value of illness perceptions to treatment adherence and illness management behaviors, with some authors finding little to no relationship (Aujla et al., 2016). Given the theoretical link to coping and behavior, these conflicting results pose a challenge to the self-regulation model. In a meta-analysis of 31 studies with different physical health conditions Dempster et al. (2015) found a strong relationship between illness perceptions and emotional health outcomes (e.g., depression and anxiety), but little evidence for the role of coping as a mediator between illness perceptions and outcomes. Again, this reinforces the need for further research given the expected relationship between illness perceptions and coping in self-regulation of health outcomes. Another venue for studying the impact of illness perceptions on coping and behavior is to study interventions aiming at correcting misconceptions. The few studied interventions, aimed at addressing illness misconceptions, have been found to have an impact in health outcomes including behavioral change (Figueiras et al., 2017).

The study of illness perceptions in healthy individuals has examined their role in prevention and early detection of particular illnesses. For example, in breast cancer risk, illness perceptions are a significant predictor of screening (Marmarà et al., 2017) and increased distress among women who are at higher risk for developing this illness (Rees et al., 2004). One important question is whether illness perceptions have the same meanings for healthy individuals. Figueiras and Alves (2007) compared the perceptions of healthy individuals using the IPQ-R for AIDS, tuberculosis, and skin cancer. They found the same factorial structure as in clinical samples, and the illness perceptions accounted for significant variance in attitudes and intentions toward the adoption of preventive behaviors. This supports the similarity of illness perceptions in healthy and sick individuals.

Overall the literature on the impacts of illness perceptions suggests that they are relevant for preventing and adjusting to illness (Figueiras and Neto, 2019). Two other aspects of the self-regulation model (Diefenbach and Leventhal, 1996) need to be considered: cultural differences and illness perceptions development. First, since particular elements differ across countries (e.g., culture, available treatments, and health information availability), national differences in illness perceptions of particular diseases are expected. The few existing studies present a mixed picture, with either significant differences (Bean et al., 2007) or minor differences (Kaptein et al., 2013) across cultural contexts. Secondly, illness perceptions are not expected to be static. This is anticipated from the original model (Leventhal et al., 2003) that assumes illness perceptions are informed by the appraisal of the consequences of the patient coping strategies (See Figure 1). This feedback cycle (i.e., the effect of the consequences of personal behavior in illness perceptions) suggests that illness perception formation is an iterative process. This process is also influenced by factors, such as the response to treatment, illness progression, and evolution on the shared representations of a given illness. Research that supports this assumption comes from the evolution of illness perceptions with the course of the illness. Significant trends have been found for particular illness perceptions during the course of diabetes (Fortenberry et al., 2014), cancer (De Castro et al., 2012), and patients undertaking hemodialysis (Tasmoc et al., 2013).

With the emergence of COVID-19, two public health goals become particularly relevant to manage the pandemic. First, the promotion of behavior change toward protective behaviors (e.g., hand-washing, social distancing). Second, to help establish the conditions for an emotional reaction (e.g., stress) within a normal range. The reviewed research suggests that illness perceptions may play an important role in the emotional and behavioral reaction to a particular illness. Therefore, understanding illness perceptions of COVID-19 may have relevant consequences for reaching these public health goals and developing public health measures, including health-promoting campaigns and their context-specific adjustments (e.g., in school settings). In the present study, we investigate illness perceptions, their predictors, and impacts across a large number of European countries.

The goals of the present study are to (1) study the development of illness perceptions across time (absolute and relative to the progression of the illness in each country); (2) understand the effect of demographic, risk and personal contact with COVID-19 (e.g., having been infected with COVID-19) on illness perceptions; and (3) assess the impacts of illness perceptions regarding general stress and preventive behaviors (e.g., hand-washing). All of these goals are studied considering the hierarchical structure of the data, with individuals nested in countries. We expect that the differences in culture and in severity of the pandemic in different countries will contribute to explaining the results.

## Materials and Methods

### Participants

The present study was part of a larger project, the COVID-19 IMPACT project (https://ucy.ac.cy/acthealthy/en/covid-19-impact-survey), which is an international online survey conducted in 78 countries/regions worldwide exploring the behavioral and psychological impacts of COVID-19. For the present study, only European countries with more than 100 participants were included in the analyses. The inclusion criteria were age of at least 18 years and the ability to read one of the 12 languages of the project (English, Finnish, French, German, Greek, Hungarian, Italian, Latvian, Polish, Portuguese, Romanian, and Spanish). There were no other exclusion criteria. The final sample size was 7,032.

Participants from 16 countries accepted to participate: Austria, Cyprus, Finland, France, Germany, Greece, Hungary, Ireland, Italy, Latvia, Poland, Portugal, Romania, Spain, Switzerland, and United Kingdom. Most participants were female (5,529; 78.6%), approximately one-fifth were males (1,479; 21.0%), and a small minority identified as other (24; 0.3%). The mean age was 37.9 years (SD = 13.3), and 484 (6.9%) participants were older than 60 years—the considered threshold for age-related risk (Williamson et al., 2020). With respect to education level, participants presented the following: a master or other postgraduate degree (2,648; 37.7%), a college/university degree (1,800; 25.6%), were attending college/university (953; 13.6%), had a high school degree (742; 10.6%), a Ph.D. (629; 8.9%), other education (207; 2.9%), or primary education (52; 0.7%).

Most participants reported little personal contact with COVID-19: most indicated that they had not been infected with COVID-19 (6,132; 87.2%), a small minority reported they were infected (67; 1.0%) and the rest had symptoms but were unsure (833; 11.8%). Similar patterns were found for partner infection rates (not infected: 6,382, 90.8%; infected: 53, 0.8%; unsure: 556, 7.9%) and infection rates of other significant persons (not infected: 5,946, 84.6%; infected: 448, 6.4%; unsure: 638, 9.1%).

### Measures

#### Illness Perceptions

Illness perception were measured using the Brief Illness Perception Questionnaire (IPQ), which was developed to assess the illness perceptions as proposed in the self-regulation model (Weinman et al., 1996). The Brief IPQ is a reduced version of the revised illness perception questionnaire for a specific disease, using eight questions in which each represents a dimension of disease perception: consequences, timeline, personal control, treatment control, identity, concern, understanding, and emotional response. The questions can be thought to depict cognitive (e.g., How much do you think existing treatments help patients with COVID-19?) or emotional illness representations (e.g., How much does COVID-19 affect you emotionally–e.g., makes you sad, angry, scared?). Each question is answered in a semantic differential scale, ranging from 0 to 10, on the importance that each dimension represents to the patient. The identity item was not included because it referred to the experience of having the illness. Higher scores reflect more negative illness perceptions. There are three inverted items (personal control, treatment control, and understanding). In the present paper, the results of these items are presented inversely to ease the interpretation. Therefore, higher scores in these items reflect a lack of personal control, treatment control, and understanding. As in other studies with non-clinical samples (Figueiras and Alves, 2007), the items were adapted to healthy individuals' perceptions. This instrument has been used widely and has shown good psychometric properties (Broadbent et al., 2006, 2015).

#### Time Variables

Time was considered in two ways: chronological and adjusted time. Chronological time refers to the number of days since the first official COVID-19-related death in Europe (in France) –February 15, 2020 (day one). Time was recoded from the timestamp date of the survey form. Considering that the epidemiological evolution of the COVID-19 pandemic was different in each country, the variable adjusted time was also created. Adjusted time refers to the number of days after the detection of the 100th case (day one). This date was considered the beginning of the pandemic in each country given that the initial cases were sporadic and mainly imported. Adjusted time, unlike chronological time, is country-specific. To avoid eventual negative values, the first day corresponds to 100. Data about the accumulated number of cases for each country were taken from the official data of the European Center for Disease Prevention and Control (ECDC, 2020). The data from the ECDC come from national agencies responsible for health statistics.

#### Predictors

Two groups of predictors were considered: sociodemographic characteristics and personal contact with COVID-19. With respect to sociodemographic characteristics, we considered age, gender, and educational status. It is important to mention that age and gender are also relevant risk factors for COVID-19 (Williamson et al., 2020). Age was recoded into younger vs. older than 60 years; participants older than 60 years were considered to be at greater risk. This threshold was chosen to balance the need for a significant number of participants and a significant higher risk of complications and death from COVID-19. There were three items related to personal contact with COVID-19. Participants were asked to report whether they, their partners, or a significant other had been diagnosed with COVID-19. They could respond yes, no, and unsure.

#### Outcomes

Two types of outcomes were studied: stress and COVID-19 preventive behaviors. Stress was assessed using the Perceived Stress Scale (PSS, 32). The PSS is a 10-item questionnaire assessing an individual's appraisal of how stressful life situations are. Items ask about people's feelings and thoughts during the last week and are scored on a 5-point Likert-type scale, ranging from 0 = never to 4 = very often. Total scores are obtained by reversing the scores on the four positively worded items (items 4, 5, 7, and 8), and then adding all 10 items. The total scores range from 0 to 40, with higher scores indicating greater overall stress.

COVID-19 preventive behaviors were assessed with three questions referring to social distance (personal distance when going out), self-isolation (following self-isolation and travel restrictions suggested by national guidelines), and hand-washing. The answer to these questions followed a semantic differential scale ranging from 0 (never) to 10 (all of the time).

### Procedure

Ethics approval was obtained from the Cyprus National Bioethics Committee (ref.: EEBK EΠ 2020.01.60) followed by site approvals from different research teams involved in data collection. All participants provided informed consent before completing the online survey in Google Survey format. Data were collected for 2 months between 7th April and 7th June 2020.

The online survey was distributed using a range of methods. Universities emailed the online survey to students and academic staff and posted the survey link to their websites. In addition, and in order to broaden the sample to older age groups and those with different sociodemographic characteristics, the survey was disseminated in the local press (e.g., newspapers, newsletters, radio stations), in social media (e.g., Facebook), in professional networks, local hospitals, and health centers, professional groups' email lists (e.g., teachers, engineers, psychologists, government workers, churches, musicians, etc).

### Data Analysis

The analytic plan was based on multilevel modeling due to the clustered structure of the data, which means individuals were nested within countries. By recognizing the non-independence of the observations, these models provide, for instance, more accurate estimations of standard errors than traditional linear regression models with residual variance being divided into between-country residuals (effects representing country elements affecting individuals) and within-country residuals (participant-level residuals) (Steele, 2010).

The analysis started by exploring multilevel correlations between illness perceptions, time variables, predictors and outcomes, providing Pearson *r* values for within and between countries. The cut-off values used for interpretation were: the association was considered weak for *r* values <0.30, moderate when *r* values were between 0.30 and 0.50, and strong whenever *r* values were higher than 0.50 (Cohen, 1992). Descriptive statistics (means and standard deviations) were also computed.

Growth curve models were estimated using multilevel modeling to check the change in illness perceptions according to chronological and adjusted time. Models had two levels illustrating participants (level-1) nested within countries (level-2). First, the optimal function to be adjusted to health trajectories was estimated. We started with an intercept-only model (no growth model), which was expanded to incorporate linear and quadratic functions. The results were interpreted for the most adequate model function (Curran et al., 2010). Variance at the individual and country-level was decomposed by calculating the intraclass correlation coefficient (ICC). Because the estimated models were nested models, likelihood ratio tests were computed (Raudenbush and Bryk, 2020) with the Akaike information criterion (AIC), and the Bayesian information criterion (BIC) indices were also used to assess model fit. When models were compared, those with a better fit present lower levels of AIC and BIC (Burnham and Anderson, 2004).

Additional multilevel models were estimated not only to identify whether sociodemographic variables and personal contact with COVID-19 contributed to explaining illness perceptions but also to evaluate if illness perceptions predicted COVID-19 preventive behaviors and stress.

For each model, unstandardized estimates (*B*), and 95% confidence intervals (CI) were computed. Parameters were significant when the 95% CI did not include 0. Following Lorah (2018) recommendations, the ICC for random effects and standardized regression coefficients (β) for fixed effects were computed as effect size measures. A maximum likelihood estimator was applied.

Multilevel modeling analyses were performed using *psych* (Revelle, 2018) and *lme4* packages (Bates et al., 2015), while effect sizes were estimated with the *sjstats* package (Lüdecke, 2020). All packages were designed for the R environment (R Core Team, 2019). Additional descriptive statistics analyses were performed using SPSS (v.26, SPSS Inc., Chicago, IL).

## Results

### Descriptive and Correlational Analyses

The mean scores found for the illness perceptions were as follows (N = 7 032): personal control 3.4 (*SD* = 2.20), consequences 7.4 (*SD* = 2.25), timeline 6.6 (*SD* = 1.80), treatment control 4.1 (*SD* = 2.14), concern 6.6 (*SD* = 2.44), understanding 2.7 (*SD* = 1.95), emotional response 6.3 (*SD* = 2.51), and total score 37.11 (*SD* = 7.729). If we consider the middle of the scale of the illness perception items, this means that in the community, with respect to cognitive representations, people tend to perceive higher consequences and duration of COVID-19. On the other hand, participants tend to believe they have good understanding, personal control and believe in the effectiveness of the existing treatments. Concerning emotional representations, the participants tended to express concern and a negative emotional response.

Participants' average stress level was 16.7 (*SD* = 7.46), which is considered at the low end of moderate stress (Cohen, 1988). With respect to the adherence to protective measures (rage: 0–10), the participants reported: maintain social distance 8.9 (*SD* = 1.49), self-isolation according to national guidelines 9.0 (*SD* = 1.71), and hand-washing 9.1 (*SD* = 1.39). Table 1 presents the considered outcomes across countries.

**Table 1:** Means (and Standard Deviations) for the outcome variables across country.

**Country**	**Illness perceptions**							**Stress**	**Risk behaviors**		
	**Pers. Control**	**Conseq**.	**Timel**.	**Treat. Control**	**Concern**	**Underst**.	**Emot. Resp**.	**PSS total**	**Social distance**	**Self-isolation**	**Handw**
Austria (*N* = 368)	3.5 (2.1)	6.4 (2.3)	6.5 (1.6)	4.0 (2.2)	5.0 (2.3)	2.8 (2.0)	5.6 (2.4)	15.8 (6.6)	8.8 (1.4)	9.2 (1.6)	9.0 (1.6)
Cyprus (*N* = 957)	2.2 (1.9)	7.9 (2.1)	7.8 (1.8)	4.2 (2.1)	7.3 (2.3)	1.9 (1.7)	6.8 (2.5)	17.6 (7.5)	8.7 (1.7)	9.2 (1.7)	9.2 (1.5)
Finland (*N* = 157)	3.7 (2.0)	7.1 (2.0)	6.1 (1.5)	3.2 (1.8)	6.4 (2.0)	3.0 (1.7)	6.1 (2.4)	16.5 (6.7)	8.6 (1.2)	9.1 (1.6)	9.4 (1.2)
France (*N* = 313)	4.1 (2.3)	7.0 (2.3)	6.5 (1.7)	4.3 (2.2)	6.0 (2.4)	3.3 (2.1)	5.9 (2.6)	15.8 (7.7)	9.0 (1.5)	9.2 (1.4)	8.6 (1.7)
Germany (*N* = 279)	3.9 (2.2)	7.0 (2.2)	6.3 (1.6)	3.7 (2.2)	5.7 (2.4)	3.1 (2.0)	6.0 (2.4)	16.8 (6.6)	8.5 (1.5)	8.7 (2.2)	8.9 (1.4)
Greece (*N* = 270)	2.1 (1.8)	7.6 (2.1)	7.8 (1.7)	4.3 (2.2)	7.3 (2.1)	2.4 (1.9)	6.6 (2.4)	16.7 (7.2)	8.3 (1.7)	8.8 (1.9)	9.2 (1.3)
Hungary (*N* = 273)	2.7 (2.0)	6.9 (2.4)	5.9 (1.8)	4.6 (2.0)	4.8 (2.5)	2.7 (2.1)	5.8 (2.6)	16.9 (7.5)	8.3 (1.9)	7.6 (2.7)	9.3 (1.4)
Ireland (*N* = 414)	3.7 (2.1)	7.6 (1.9)	6.6 (1.6)	4.5 (2.2)	6.6 (2.2)	2.3 (1.7)	6.4 (2.3)	15.9 (7.8)	9.3 (1.1)	9.4 (1.2)	9.1 (1.3)
Italy (*N* = 962)	3.8 (2.2)	8.4 (1.7)	6.9 (1.6)	3.5 (1.9)	7.3 (2.0)	3.0 (1.9)	6.6 (2.3)	16.7 (6.5)	9.4 (1.1)	9.5 (1.1)	9.2 (1.3)
Latvia (*N* = 1,285)	3.7 (2.2)	7.5 (2.3)	5.8 (1.8)	4.2 (2.3)	6.7 (2.5)	2.6 (2.0)	6.4 (2.6)	17.7 (8.4)	8.8 (1.4)	8.7 (1.7)	9.4 (1.2)
Poland (*N* = 135)	4.3 (2.0)	7.9 (2.2)	6.5 (1.6)	4.6 (2.0)	6.0 (2.4)	3.2 (1.9)	6.3 (2.3)	18.5 (6.7)	8.2 (1.9)	8.7 (1.9)	8.9 (1.6)
Portugal (*N* = 334)	3.2 (1.9)	7.6 (2.0)	6.9 (1.6)	3.8 (2.1)	8.0 (1.9)	2.3 (1.9)	6.5 (2.4)	14.6 (7.4)	9.0 (1.4)	9.3 (1.6)	8.9 (1.4)
Romania (*N* = 339)	3.9 (2.3)	7.1 (2.3)	6.0 (1.8)	4.0 (2.1)	6.5 (2.4)	2.8 (1.9)	6.0 (2.7)	17.3 (7.4)	8.8 (1.5)	8.9 (1.7)	9.5 (1.1)
Spain (*N* = 296)	3.5 (2.1)	7.3 (2.2)	7.1 (1.6)	3.9 (2.0)	8.0 (1.9)	2.6 (1.9)	6.9 (2.3)	16.0 (7.9)	9.0 (1.4)	9.5 (1.1)	9.0 (1.5)
Switzerl. (*N* = 550)	3.8 (1.9)	6.0 (2.4)	6.1 (1.5)	3.8 (2.0)	5.2 (2.3)	3.0 (1.9)	5.4 (2.5)	16.3 (6.8)	8.5 (1.5)	8.7 (1.8)	8.8 (1.5)
U.K (*N* = 100)	3.9 (2.2)	7.4 (2.1)	6.3 (1.7)	4.8 (2.0)	6.5 (2.3)	2.5 (1.6)	6.5 (2.3)	17.5 (8.0)	9.0 (1.4)	9.1 (1.7)	9.0 (1.5)

*Complete names for the variables: illness perceptions (personal control, consequences, timeline, treatment control, concern, understanding, emotional response), risk behaviors (social distance, self-isolation, hand-washing)*.

In regard to the time variables considered, chronological time ranged from 46 to 104 days (*M* = 68.9; *SD* = 11.15). This corresponds to an adjusted time ranging from 109 to 192 (*M* = 144.1; *SD* = 15.48), or, alternatively, initiating 9 days after the 100th case. These time ranges provide information as to when, in the epidemiological evolution of the pandemic, were the study variables being measured. Within and between countries Pearson correlations are presented in Table 2. Overall, results suggest no difference at the level of country.

**Table 2:** Multilevel correlations between illness perceptions, preventive behaviors, and stress.

	**1**	**2**	**3**	**4**	**5**	**6**	**7**	**8**	**9**	**10**	**11**	**12**	**13**
1 Chronological time	-	1	−0.06	−0.05	0.02	0.01	−0.05	0.01	−0.04	−0.02	0.01	−0.01	0.04
2 Adjusted time	0.66	–	−0.06	−0.05	0.02	0.01	−0.05	0.01	−0.04	−0.02	0.01	−0.01	0.04
3 Social distance	−0.11	0.32	–	0.44	0.35	−0.03	0.07	0.06	0.22	0.07	−0.19	−0.05	−0.13
4 Self isolation	−0.19	0.24	0.76	–	0.23	−0.01	0.05	0.05	0.17	0.04	−0.13	−0.04	−0.07
5 Hand-washing	−0.09	−0.40	0.06	−0.23	–	−0.04	0.07	0.04	0.17	0.08	−0.16	−0.06	−0.10
6 Stress (PSS)	−0.37	−0.50	−0.28	−0.41	0.66	–	0.32	0.14	0.22	0.52	0.15	0.06	0.11
7 Consequences	−0.58	0.28	0.55	0.52	0.35	0.23	–	0.15	0.32	0.48	0.04	0.01	0.01
8 Timeline	−0.37	−0.20	0.07	0.55	−0.27	−0.27	0.47	–	0.28	0.24	−0.03	0.05	−0.04
9 Concern	−0.39	−0.22	0.51	0.63	0.20	−0.07	0.81	0.55	–	0.53	−0.04	−0.02	−0.07
10 Emotional response	−0.46	−0.36	0.41	0.52	0.31	0.18	0.90	0.59	0.92	–	0.07	0	0
11 Personal control	0.29	0.56	0.38	0.05	−0.10	0	−0.23	−0.74	−0.29	−0.41	–	0.27	0.24
12 Treatment control	−0.04	−0.53	−0.35	−0.40	0.11	0.29	−0.08	−0.03	−0.15	0.06	−0.3	–	0.15
13 Understanding	0.26	0.68	0.10	−0.14	−0.24	−0.04	−0.35	−0.59	−0.49	−0.60	0.81	−0.45	–

*Correlation within country above the diagonal. Correlation between country below the diagonal. Correlations were considered small (r < 0.30), moderate (r values between 0.30 and 0.50), and strong (r > 0.50) (33)*.

### Illness Perceptions Trajectories

The fit indices and likelihood ratio tests for each illness perception and growth function are shown in Table 3. Non-significant chi-square statistics were found for emotional response, personal and treatment control, suggesting the intercept-only model was the best option for these perceptions (no growth model). For timeline trajectories, the quadratic function was the most adequate, suggesting timeline average trajectory increases, but it changes at some point in time becoming curvilinear (see Table 4). In regard to the remaining illness perceptions, trajectories were best modeled by a linear function. For linear growth models, the results suggested higher levels of understanding as time increased, with the opposite occurring for consequences and concern (Tables 3, 4). Examples of graphical representations of the functions found for the trajectories of illness perceptions with higher ICC values are included in the Supplementary Materials.

**Table 3:** Model fit information regarding growth curves optimal functions for chronological and adjusted time.

	**Intercept-only**		**Linear**		**Quadratic**		**df, χ^2^_diff_**	**Model comparison**
	**AIC**	**BIC**	**AIC**	**BIC**	**AIC**	**BIC**		
**Chronological time**								
Consequences	30,871	30,891	30,850	30878	30,860	30,928	1, 22.6924^***^	Intercept-only vs. linear
Timeline	27,201	27,222	27,103	27,230	27,188	27,256	6, 26.9896^***^	Linear vs. quadratic
Emotional response	32,728	32,749	32,727	32,754	32,732	32,801	1, 3.0452	Intercept-only vs. linear
Personal control	30,506	30,527	30,508	30,535	30,516	30,585	1, 0.3587	Intercept-only vs. linear
Treatment control	30,514	30,535	30,515	30,542	30,526	30,594	1, 1.7232	Intercept-only vs. linear
Concern	31,581	31,602	31,571	31,599	31,577	31,645	1, 12.0645^**^	Intercept-only vs. linear
Understanding	29,102	29,122	29,094	29,121	29,098	29,167	1, 9.9518^**^	Intercept-only vs. linear
**Adjusted time**								
Consequences	30,871	30,891	30,850	30,878	30,860	30,929	1, 22.3831^***^	Intercept-only vs. linear
Timeline	27,201	27,222	27,203	27,230	27,185	27,254	6, 29.3879^***^	Linear vs. quadratic
Emotional response	32,728	32,749	32,727	32,754	32,735	32,804	1, 3.1141	Intercept-only vs. linear
Personal control	30,506	30,527	30,508	30,535	30,517	30,585	1, 0.7783	Intercept-only vs. linear
Treatment control	30,514	30,535	30,513	30,541	30,523	30,591	1, 2.8344	Intercept-only vs. linear
Concern	31,581	31,602	31,572	31,599	31,576	31,644	1, 11.8417^**^	Intercept-only vs. linear
Understanding	29,102	29,122	29,090	29,118	29,098	29,166	1, 13.1775^**^	Intercept-only vs. linear

*** *p < 0.001*.

** *p < 0.01*.

**Table 4:** Estimates for intercept-only models and effects of time on illness perceptions.

		***B***	**β**	**95% CI (*B*)**	**ICC**
**Chronological time**					
Consequences	Linear function	−0.82	−0.07	[−1.16, −0.48]	0.06
Timeline	Quadratic function	2.08	0.04	[1.07, 3.09]	0.11
Emotional response	Intercept-only	6.24	0.03	[6.01, 6.46]	0.03
Personal control	Intercept-only	3.50	0.03	[3.18, 3.82]	0.08
Treatment control	Intercept-only	4.09	0.02	[3.89, 4.30]	0.03
Concern	Linear function	−0.63	−0.05	[−0.99, −0.28]	0.15
Understanding	Linear function	0.47	0.05	[0.18, 0.77]	0.03
**Adjusted time**					
Consequences	Linear function	−1.14	−0.09	[−0.13, −0.06]	0.06
Timeline	Quadratic function	2.89	0.06	[1.68, 4.13]	0.1
Emotional response	Intercept-only	6.24	0.03	[6.01, 6.46]	0.03
Personal control	Intercept-only	3.50	0.03	[3.18, 3.82]	0.08
Treatment control	Intercept-only	4.09	0.02	[3.89, 4.30]	0.03
Concern	Linear function	−0.89	−0.07	[−1.39, −0.38]	0.15
Understanding	Linear function	0.76	0.07	[0.35, 1.17]	0.03

### Predictors of Illness Perceptions

Table 5 presents the results for the multilevel models exploring the role of sociodemographic variables in explaining illness perceptions. Specifically, age of at least 60 years was negatively associated with perceived consequences, emotional response, and personal control perceptions, and positively related to timeline and concern. Female gender revealed an association with higher perceived consequences, timeline, emotional response, concern, treatment control, and lower understanding. Higher education levels were associated with higher perceived understanding when compared to primary education level. For participants diagnosed with COVID-19, more negative consequences were perceived, and when their partners were diagnosed, higher levels of personal control were identified.

**Table 5:** Multilevel modeling Regression coefficients, confidence intervals and ICC values for illness perceptions predictors.

**Predictors**	**Consequences (ICC = 0.07)**			**Timeline (ICC = 0.12)**			**Emotional response (ICC = 0.03)**			**Personal control (ICC = 0.08)**		
	***B***	**95% CI (*B*)**	**β**	***B***	**95% CI (*B*)**	**β**	***B***	**95% CI (*B*)**	**β**	***B***	**95% CI (*B*)**	**β**
Intercept	7.86	[6.88, 8.34]		6.61	[5.83, 7.39]		5.74	[4.97, 6.51]		4.24	[2.62, 4.02]	
Age	**−0.23**	**[−0.45**, **−0.02]**	**−0.1**	**0.21**	**[0.04, 0.37]**	**0.11**	**−0.25**	**[−0.50**, **−0.00]**	**−0.1**	**−0.47**	[−0.68, −0.26]	−0.22
Gender	**0.59**	**[0.45, 0.73]**	**0.26**	**0.15**	**[0.04, 0.25]**	**0.08**	**1.07**	**[0.91, 1.23]**	**0.43**	−0.05	[−0.18, 0.09]	−0.02
Education 1	0.19	[−0.48, 0.86]	0.08	−0.04	[−0.56, 0.48]	−0.00	−0.19	[−0.94, 0.57]	−0.07	−0.02	[−0.67, 0.62]	−0.01
Education 2	0.14	[−0.53, 0.80]	0.06	0.08	[−0.43, 0.59]	0.04	−0.19	[−0.94, 0.57]	−0.07	0.06	[−0.59, 0.70]	0.03
Education 3	0.16	[−0.49, 0.82]	0.07	0.16	[−0.35, 67]	0.09	−0.24	[−0.98, 0.50]	−0.1	0.12	[−0.51, 0.76]	0.06
Education 4	0.11	[−0.55, −0.76]	0.05	0.03	[−0.47, 0.54]	0.02	−0.54	[−1.28, 0.20]	−0.21	0.29	[−0.34, 0.92]	0.13
Education 5	−0.05	[−0.75, 0.56]	−0.00	0.05	[−0.47, 0.57]	0.03	−0.48	[−1.25, 0.28]	−0.19	0.27	[−0.38, 0.92]	0.13
Education 6	0.13	[−0.60, 0.86]	0.06	0.18	[−0.38, 0.75]	0.1	−0.08	[−0.90, 0.74]	−0.03	−0.03	[−0.72, 0.68]	−0.01
COVID self	**1.08**	**[0.32, 1.84]**	**0.48**	−0.47	[−1.06, 0.11]	−0.3	0.61	[−0.25, 1.46]	0.24	0.29	[−1.02, 0.44]	−0.13
COVID par.	0.11	[−0.97, 0.75]	−0.1	0.61	[−0.06, 1.27]	0.34	−0.17	[−1.14, 0.80]	−0.07	**−1.21**	**[0.39, 2.04]**	**0.56**
COVID oth.	−0.23	[−0.01, 0.47]	0.1	0.10	[−0.08, 0.29]	0.06	0.02	[−0.25, 0.29]	0.01	0.00	[−0.25, 0.23]	0.00
	**Treatment control (ICC=0.04)**			**Concern (ICC=0.15)**			**Understanding (ICC=0.04)**					
Predictors	*B*	95% CI (*B*)	β	*B*	95% CI (*B*)	β	*B*	95% CI (*B*)	β			
Intercept	3.76	[3.09, 4.43]		5.73	[4.90. 2.27]		3.26	[2.66, 3.86]				
Age	0.00	[−0.21, 0.22]	0.00	**0.41**	**[0.18, 0.63]**	**0.17**	0.04	[−0.16, 0.23)	0.02			
Gender	**−0.01**	**[−0.28**, **−0.01]**	**−0.1**	**0.76**	**[0.62, 0.90]**	**0.31**	**0.12**	**[0.00, 0.24]**	**0.06**			
Education 1	0.21	[−0.45, 0.86]	0.1	0.03	[−0.66, 0.73]	0.01	−0.57	[−1.16, 0.02]	−0.3			
Education 2	0.24	[−0.41, 0.88]	0.11	0.26	[−0.43, 0.95]	0.11	**−0.66**	**[−1.24**, **−0.07]**	**−0.34**			
Education 3	0.31	[−0.33, 0.95]	0.15	0.30	[−0.38, 0.98]	0.13	**−0.72**	**[−1.29**, **−0.14]**	**−0.37**			
Education 4	0.45	[−18, 1.09]	0.21	0.07	[−0.61, 0.75]	0.03	−55	[−1.12, 0.02]	−0.29			
Education 5	0.60	[−0.06, 1.26]	0.28	−0.07	[−0.76, 0.63]	−0.03	**−0.72**	**[−1.31**, **−0.13]**	**−0.38**			
Education 6	0.33	[−0.38, 1.04]	0.15	0.52	[−0.23, 1.27]	0.22	−0.59	[−1.23, 0.04]	−0.31			
COVID self	−0.38	[−1.12, 0.36]	−0.2	0.10	[−0.68, 0.89]	0.04	−0.17	[−0.83, 0.50]	−0.09			
COVID par.	0.72	[−0.12, 1.56]	0.34	−0.74	[−1.64, 0.15]	−0.31	0.04	[−0.72, 0.79]	0.02			
COVID oth.	0.05	[−0.18, 0.29]	0.03	0.06	[−0.19, 0.31]	0.02	−0.10	[−0.31, 0.11]	−0.05			

*Age (Over 60), Gender (Female), Education 1 (Highschool), Education 2 (Some), Education 3 (Graduated), Education 4 (Post-Graduated/MA), Education 5 (PhD), Education 6 (Other), COVID-19 Self (Yes), COVID-19 Partner (Yes), COVID-19 Other (Yes)*.

*For education level. The reference group is “Primary.” While for COVID-19 diagnosis the reference category is “No.” Also “No” is the reference category for Age Risk. As for Gender “Male” is the reference group. Values in bold correspond to 95% CI not including 0*.

*The 95% CI (B) for age predicting emotional response ranges between −0.496 and −0.002*.

### Illness Perception Contributions to Explain COVID-19 Preventive Behaviors and Stress

Lower ICC values were found for COVID-19 preventive behaviors and stress, suggesting similarities between countries. Social distance was linked to higher concern and personal control and lower perceived understanding and negative emotional response. Social isolation presented an association with lower perceived emotional response and higher personal control, understanding, concern and consequences. Finally, hand-washing was related to higher perceived consequences, concern, personal control and understanding. Higher standardized estimates were found for personal control and concern for all COVID-19 preventive behaviors. All illness perceptions showed a significant and positive association with stress, except for treatment control. Consequences, followed by personal control and concern, presented higher standardized estimates. These results are presented in Table 6.

**Table 6:** Multilevel modeling estimates and ICC values for COVID-19 behavioral outcomes and stress.

**Predictors**	**Social distance (ICC = 0.05)**			**Self-isolation (ICC = 0.07)**			**Hand-washing (ICC = 0.03)**			**Stress (ICC = 0.01)**		
	***B***	**95% CI (*B*)**	**β**	***B***	**95% CI (*B*)**	**β**	**B**	**95% CI (*B*)**	**β**	***B***	**95% CI CI (*B*)**	**β**
Intercept	8.44	[8.20, 8.69]		8.57	[8.29, 8.87]		8.84	[8.63, 9.05]		3.46	[2.54, 6.32]	
Consequences	0.02	[−0.00, 0.03]	0.03	**0.02**	**[0.00, 0.04]**	**0.03**	**0.02**	**[0.01, 0.04]**	**0.04**	**0.31**	**[0.24, 0.39]**	0.09
Timeline	−0.00	[−0.03, 0.02]	0	0.00	[−0.02, 0.02]	0	−0.01	[−0.03, 0.01]	−0.01	**0.12**	**[0.03, 0.21]**	0.03
Emot. respon.	**−0.03**	**[−0.04**, **−0.01]**	**−0.04**	**−0.05**	**[−0.07**, **−0.03]**	**−0.08**	0	[−0.02, 0.01]	−0.01	**1.51**	**[1.43, 1.58]**	0.51
Pers. control	**−0.11**	**[−0.13**, **−0.10]**	**−0.16**	**−0.09**	**[−0.11**, **−0.07]**	**−0.11**	**−0.09**	**[−0.10**, **−0.07]**	**−0.14**	**0.31**	**[0.24, 0.38]**	0.09
Treat. control	0.00	[−0.01, 0.02]	0.01	0.00	[−0.02, 0.02]	0	−0.01	[−0.02, 0.01]	−0.01	**0.08**	**[0.01, 0.15]**	0.02
Concern	**0.14**	**[0.12, 0.16]**	**0.23**	**0.14**	**[0.12, 0.16]**	**0.21**	**0.09**	**[0.08, 0.11]**	**0.17**	**−0.25**	**[−0.33**, **−0.17]**	−0.08
Understanding	**0.06**	**[−0.07**, **−0.04]**	**−0.07**	**−0.03**	**[−0.05**, **−0.01]**	**−0.03**	**−0.04**	**[−0.06**, **−0.02]**	**−0.06**	**0.32**	**[0.24, 0.40]**	0.08

*Values in bold correspond to 95% CI not including 0*.

## Discussion

The present study sought to investigate illness perceptions for COVID-19 and study three goals. The first goal was to understand the development of illness perceptions across time—considering chronological time and time adjusted to the epidemiological evolution of the pandemic in each county. The first consideration about these results is that chronological time and adjusted time showed the same results in terms of the direction and significance of their trends. This may suggest that individuals in Europe were reacting similarly to information from the progression of COVID-19 in other countries. The second consideration is that the magnitude of the temporal effects is small and only observed in some illness perceptions (i.e., consequences, timeline, concern, and understanding). This result needs to be interpreted considering the data gathering period—starting 46 days after the first COVID-19-related death in Europe. The small magnitude of the trend can have several interpretations. First, it may be that illness perceptions of COVID-19 were formed early in the pandemic and remained fairly stable. If so, the small magnitude of the trends would reflect the later stage of this formation. Specifically, the results suggest a linear decrease in the perceived understanding and perceived negative consequences and concern about COVID-19. The linear progression suggests a decrease in the negativity of illness perceptions over time. Second, illness perceptions may change across time as a function of the socially perceived dangerousness of COVID-19. This perception could be shaped by variables such as the perceived incidence of the condition on a given region or in a given time. If so, the current study only presents a picture of a given period, and evolution would be non-linear. The quadratic function of the trajectory of the timeline may be understood in this light. Future research, including longitudinal studies, will allow testing these alternative interpretation hypotheses, confirming either the stable or fluctuating nature of COVID-19 illness perceptions.

The second goal was to examine the predictors of the illness perceptions, across European countries. The first finding is that country showed no influence as a level of the model due to small ICC values. Different countries reflect not only cultural differences but also different epidemiological situations—at the considered time span. In any case, these results suggest a cross-national character of illness perceptions, at least for COVID-19 in Europe. As aforementioned, the cultural comparisons of illness perceptions are conflicting (Bean et al., 2007; Kaptein et al., 2013). Future research could consider countries outside of Europe or change the considered level for the analysis from individual countries to European regions (northern vs. southern; western vs. eastern).

The age risk group and gender showed a significant effect on several illness perceptions. Being considered in an age risk group was associated with illness perceptions in a mixed way. Age older than 60 years was positively associated with concern and timeline; however, concerningly, it was associated with lower perceived consequences, higher personal control, and with a better emotional response. This mixed pattern of associations may be related to general representations of old age interacting with illness perceptions (e.g., “my body is frail” vs. “I have survived so many ordeals, it is not a flu that will keep me from living”). Gender was also associated with illness perceptions in a mixed way. Female gender was associated with higher perceived personal and treatment control; however, it was associated with higher perceived consequences, timeline, negative emotional response, concern, and lower understanding. Again this could be related to general gender attitudes that associate being male with minimization of health threats. The results are consonant with findings on health-related attitudes associated with gender and age (Deeks et al., 2009), which are in agreement with the influence of general culture in the Common-sense model of self-regulation (e.g., Diefenbach and Leventhal, 1996). Given that male gender and age older than 60 years are risk factors for COVID-19, these results are unsettling. Some of the illness perceptions are linked to the risk factors in a way that is contrary to what would be desirable (i.e., higher risk, higher negativity). Understanding the specific illness perceptions that differ in these groups may inform specific focuses on health-promoting campaigns. Higher education levels, expectedly, were associated with a lower level of perceived lack of understanding—but no difference was found for the remaining perceptions. Personal contact with COVID-19 presented mixed results. For participants reporting having contracted COVID-19, higher negative consequences were perceived. However, when it was the partner that was infected with COVID-19, the participants tended to report higher levels of perceived personal control. It may be the case that while having COVID-19 may make participants realize its negative consequences, taking care of a partner with COVID-19 may foster the idea of personal control over the condition.

The third goal was to understand the impact of illness perceptions on general stress and COVID-19 preventive behaviors. Given the response scale of the IPQ items, higher scores reflect more negative illness perceptions. Concerning stress, the results were overall as expected, with all illness perceptions showing a significant and positive association with stress (i.e., all except for treatment control). The results of COVID-19 preventive behavior are less clear. As expected, higher scores in concern are associated with higher social distance, social isolation, and hand-washing. Perceived personal control is associated with higher social distance, social isolation, and hand-washing. Understanding shows a mixed result—with a association with lower social distance but with higher social isolation and hand-washing. These results show that illness perceptions explain general stress more consistently than the adoption of COVID-19 preventive behaviors. However, several points should be mentioned to caution such interpretation. First, the behavior dimensions are measured with a self-report scale, which raises questions about whether actual behavior is being measured. Second, during the period of the study, there were state-mandated guidelines (including lockdowns in some countries) to perform specific behaviors. This is unlike most conditions under which illness perceptions have been studied and raises the possibility of different determinants of this adherence. The third consideration is with respect to interpretation of the IPQ for COVID-19. Unlike other diseases in which illness perception has been studied, COVID-19 is a new condition. The participant's interpretation of items such as treatment control or understanding may be affected by the lack of scientific knowledge or consensus on the disease. It could be argued that such objective considerations are irrelevant for the consideration of illness perceptions. However, this is an important difference from most of the existing literature on familiar diseases, and it may create differences from other illnesses perceptions less dependent on such knowledge—such as concern. The implications of this consideration are two-fold. First, some of the inconsistencies among illness perceptions and other variables found in the present study may be due to interpretation issues. Second, these inconstancies may reflect illness perception formation—rendering these results an exceptional snapshot of this process.

The differential relationship between illness perceptions outcomes is similar to other studies that find that illness perceptions are better at explaining psychological dimensions than behavioral dimensions (Dempster et al., 2015; Aujla et al., 2016). Therefore, it may be advantageous to add other variables (e.g., existing barriers, self-efficacy) to illness perceptions in explaining the behavior. Risk perception has been showing promise to complement illness perception since it specifically refers to personal risk of contracting the disease. The few studied conducted for COVID-19 have shown risk perceptions to be relevant for precautionary health behavior in health professionals (Girma et al., 2020) perceived negative feeling in quarantined adolescents (Commodari and La Rosa, 2020) depression (Ding et al., 2020).

This study has several limitations. First, the cross-sectional nature of the analysis implies added care in extrapolating temporal variations or predictive relations between variables. Second, all measures were self-reported, which may introduce bias in reporting such as social desirability bias. Third, mask-wearing was not included as a preventive behavior. During the period of the design and implementation of the study, the recommendation of wearing masks was not so widespread. Fourth, despite the large number of participants and the effort to have multiple recruitment sources, the sample is not representative of the population. This opens the possibility of selection bias affecting the results. Concerning the analysis, despite the ability to adjust growth models to illness perceptions, standardized estimates and ICC values were small and AIC and BIC values were quite similar between models, suggesting a residual impact of both time and country, thereby requiring cautiousness in its interpretation. Nevertheless, these findings are aligned with other research addressing health outcomes in secondary schools, where strong variation in ICCs occurs, with some values lower than 0.10 (Shackleton et al., 2016). In addition, different operationalizations of time may lead to different results, reinforcing the need to interpret results cautiously.

Irrespective of the care that should be taken given the nature of the study and the recent character of COVID-19, this study has several implications. First it supports illness perceptions as a relevant concept in understanding disease—even with non-clinical samples. The results of this study may, for example, be used to inform health promotion campaigns for particular themes that may be relevant for particular risk groups—namely, in targeting particular representations. Second, the results suggest that time may play a role in explaining perceptions, with some perceptions revealing a higher predisposition to be temporally modeled. Despite the need for research to clarify temporal evolution, knowledge of such a progression may have implications for relevant issues for pandemic management, such as reducing societal panic vs. managing public saturation and avoidance. Finally, the consideration of illness perceptions with other relevant variables may help to promote behavioral change associated with preventive measures that are required for the general public.

## Data Availability Statement

The raw data supporting the conclusions of this article will be made available by the authors, without undue reservation.

## Ethics Statement

The studies involving human participants were reviewed and approved by Cyprus National Bioethics Committee (ref.: EEBK EΠ 2020.01.60). The patients/participants provided their written informed consent to participate in this study.

## Author Contributions

DD, AN, MR, AG, MK, and AK: design of the study. DD, AN and MR: literature review and data analysis. DD, AN, AG, MK, AK, JL, MC, CN, DL, SP, SH, GP, VS, VV, LM, J-LM, AB, JA-G, M-PB, FM, SV-S, DO, RL, and BK: data gathering. DD, AN, MR, AG, MK, AK, JL, MC, CN, DL, SP, SH, GP, VS, VV, LM, J-LM, AB, JJ, M-PB, FM, SV-S, DO, RL, and BK: discussing the results. DD, AN, MR, and AK: writing up. DD, AN, MR, AG, MK, AK, JL, MC, CN, DL, SP, SH, GP, VS, VV, LM, JM, AB, JA-G, MP-B, FM, SV-S, DO, RL, and BK: reviewing and amending the paper. All authors contributed to the article and approved the submitted version.

## Conflict of Interest

The authors declare that the research was conducted in the absence of any commercial or financial relationships that could be construed as a potential conflict of interest.
