# Corrigendum: Illness perceptions of COVID-19 in Europe: predictors, impacts and temporal evolution

**DOI:** 10.3389/fpsyg.2023.1227506

**Published:** 2023-06-13

**Authors:** David Dias Neto, Ana Nunes da Silva, Magda Sofia Roberto, Jelena Lubenko, Marios Constantinou, Christiana Nicolaou, Demetris Lamnisos, Savvas Papacostas, Stefan Höfer, Giovambattista Presti, Valeria Squatrito, Vasilis S. Vasiliou, Louise McHugh, Jean-Louis Monestès, Adriana Baban, Javier Alvarez-Galvez, Marisa Paez-Blarrina, Francisco Montesinos, Sonsoles Valdivia-Salas, Dorottya Ori, Raimo Lappalainen, Bartosz Kleszcz, Andrew Gloster, Maria Karekla, Angelos P. Kassianos

**Affiliations:** ^1^ISPA - Instituto Universitário, Lisboa, Portugal; ^2^Applied Psychology Research Center Capabilities & Inclusion, Lisboa, Portugal; ^3^Faculdade de Psicologia, Universidade de Lisboa, Lisboa, Portugal; ^4^Psychological Laboratory, Faculty of Public Health and Social Welfare, Riga Stradiņš University, Riga, Latvia; ^5^Department of Social Sciences (Cyprus), School of Humanities and Social Sciences, University of Cyprus, Nicosia, Cyprus; ^6^Department of Nursing (Cyprus), Cyprus University of Technology, Limassol, Cyprus; ^7^Department of Health Sciences, European University Cyprus, Nicosia, Cyprus; ^8^The Cyprus Institute of Neurology and Genetics, The University of Nicosia Medical School, Nicosia, Cyprus; ^9^Medical University Innsbruck, Innsbruck, Austria; ^10^Department of Human and Social Sciences, Kore University Behavioral Lab (KUBeLab), Kore University of Enna, Enna, Italy; ^11^Kore University Behavioral Lab (KUBeLab), Faculty of Human and Social Sciences, Kore University of Enna, Enna, Italy; ^12^School of Applied Psychology, University College Cork, Cork, Ireland; ^13^School of Psychology (Ireland), University College Dublin, Dublin, Ireland; ^14^LIP/PC2S, University Grenoble Alpes, Grenoble, France; ^15^Department of Psychology, Babes-Bolyai University, Cluj-Napoca, Romania; ^16^Department of Biomedicine, Biotechnology and Public Health, University of Cadiz, Cádiz, Spain; ^17^Instituto ACT, Madrid, Spain; ^18^Department of Psychology, European University of Madrid, Madrid, Spain; ^19^Department of Psychology and Sociology, University of Zaragoza, Zaragoza, Spain; ^20^Vadaskert Child and Adolescent Psychiatric Hospital, Budapest, Hungary; ^21^Department of Psychology, University of Jyväskylä, Jyväskylä, Finland; ^22^Private Practice, Warsaw, Poland; ^23^Division of Clinical Psychology & Intervention Science, Department of Psychology, University of Basel, Basel, Switzerland; ^24^Department of Psychology, University of Cyprus, Nicosia, Cyprus; ^25^Department of Applied Health Research, University College London (UCL), London, United Kingdom

**Keywords:** illness perceptions, COVID-19, common sense model, illness representations, stress

In the published article, there was an error in the **Funding** statement as published. The reference for the funding was missing. The correct **Funding** statement appears below.

