# Supplementary material for: Illness Perceptions of COVID-19 in Europe: Predictors, Impacts and Temporal Evolution
Source: Front Psychol. 2021 Apr 14;12:640955. doi: 10.3389/fpsyg.2021.640955 (PMC8079952; doi:10.3389/fpsyg.2021.640955)

Supplementary Material

Graphical representations of the functions found country distribution and for the trajectories of illness perceptions with higher ICC


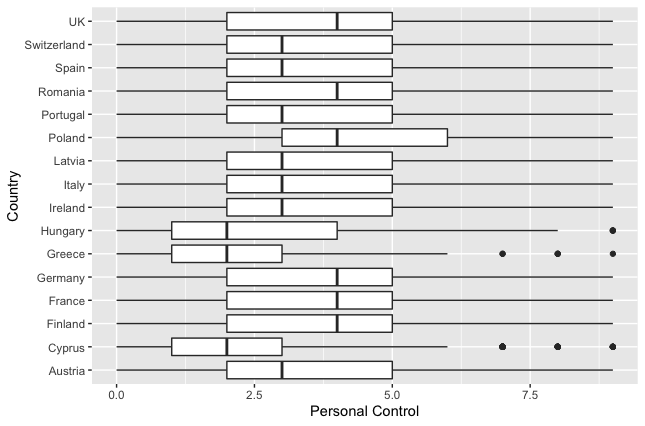


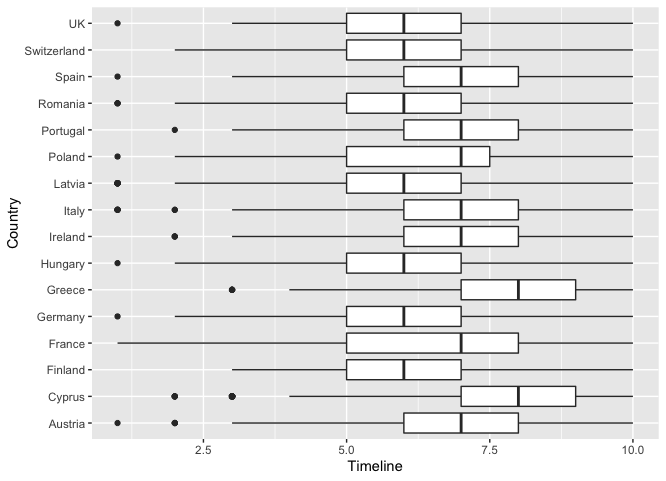


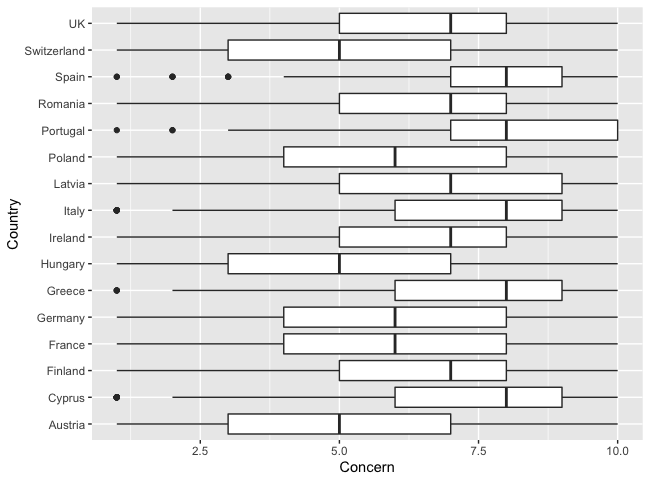


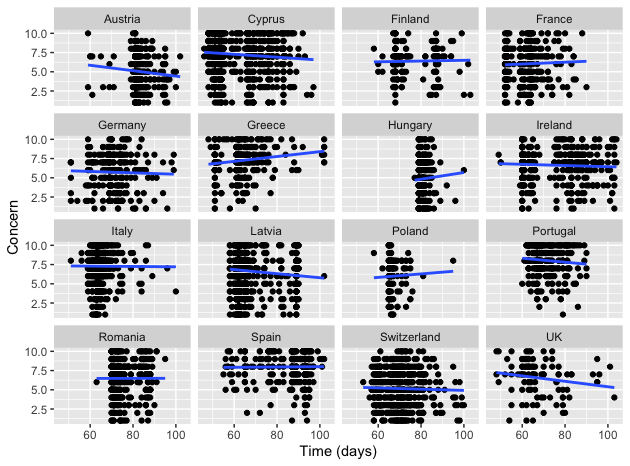


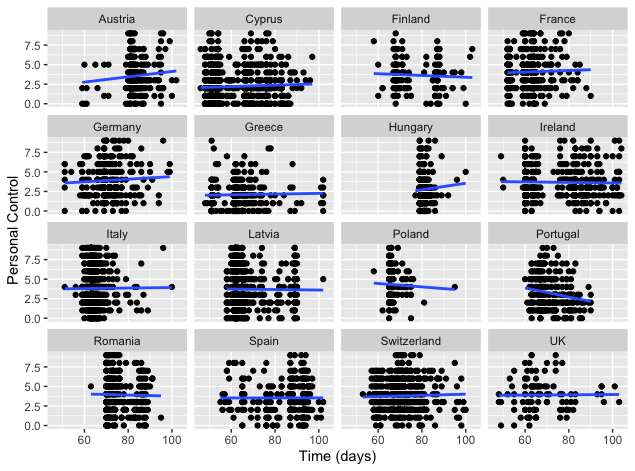


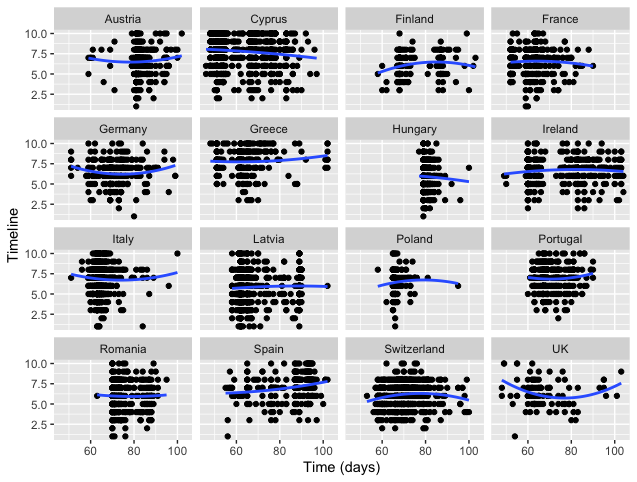

Supplement: Supplementary file 1 [file Data_Sheet_1.docx]
